# Triple-modality diagnosis of paucibacillary pulmonary tuberculosis: comparative accuracy of GeneXpert MTB/RIF, TB-DNA PCR, and T-SPOT.TB

**DOI:** 10.3389/fmed.2026.1819718

**Published:** 2026-09-07

**Authors:** Xiaohong Ji, Yu Yi, Hongyan Zhou, Xiao Chen, Huilin Zhong, Zijun Ji, Ting Guo

**Affiliations:** 1 Department of Infectious Diseases, The Second Affiliated Hospital of Yichun University, Yichun, Jiangxi, China; 2 Department of Laboratory Medicine, The Second Affiliated Hospital of Yichun University, Yichun, Jiangxi, China; 3 Yichun Vocational Technical College, Yichun, Jiangxi, China; 4 Jiangxi Vocational College of Technology, Ganzhou, Jiangxi, China; 5 The Second Affiliated Hospital of Yichun University, Yichun, Jiangxi, China

**Keywords:** balanced accuracy, diagnostic accuracy, GeneXpert MTB/RIF, IGRA, paucibacillary, pulmonary tuberculosis, TB-DNA PCR, T-SPOT.TB

## Abstract

**Background:**

Paucibacillary pulmonary tuberculosis (PTB) is diagnostically challenging because molecular assays are inherently insensitive at sub-threshold bacillary loads. The incremental value of T-SPOT.TB—a host immune-response assay (an ELISPOT-based interferon-γ release assay, IGRA)—combined with molecular testing in this setting is not well established.

**Methods:**

In this single-center, STARD 2015-compliant prospective diagnostic-accuracy study at a regional general hospital (January 2021–December 2023), adults with suspected smear-negative PTB underwent simultaneous GeneXpert MTB/RIF, in-house IS6110 TB-DNA real-time PCR, and T-SPOT.TB. Each participant was classified as PTB or non-PTB by a blinded three-member panel using a reference standard with two PTB-confirmation routes—microbiological (positive mycobacterial culture) or clinical (compatible clinicoradiological presentation plus documented response to standard anti-TB therapy at 6 months); participants meeting neither route were non-PTB. Performance was quantified by sensitivity, specificity, predictive values, likelihood ratios, and balanced accuracy ([sensitivity + specificity]/2). Paired comparisons used the McNemar exact test with Bonferroni correction. No a priori sample-size calculation was performed; analyses are exploratory.

**Results:**

Among 78 participants [PTB *n* = 44 (microbiological *n* = 33, clinical *n* = 11); non-PTB *n* = 34], T-SPOT.TB had the highest individual sensitivity (79.5%; 95% CI 65.5–88.8%) and balanced accuracy (0.780; 0.681–0.872), versus GeneXpert (29.5%; 0.618) and TB-DNA PCR (31.8%; specificity 100%; 0.659). Under a parallel (any-positive) rule, TB-DNA PCR + T-SPOT.TB showed the highest balanced accuracy observed in this cohort (0.826; 0.734–0.907; sensitivity 88.6%, specificity 76.5%; the sensitivity gain over T-SPOT.TB alone did not reach statistical significance, exact McNemar p = 0.125). The triple combination did not improve discrimination (0.796). T-SPOT.TB was the sole positive assay in 21/44 (47.7%) confirmed PTB cases.

**Conclusion:**

In this exploratory single-center cohort, T-SPOT.TB contributed unique diagnostic information (47.7% exclusive detection) not captured by molecular assays. TB-DNA PCR + T-SPOT.TB showed the highest observed balanced accuracy, but, given the single-center design and absence of a priori powering, this is hypothesis-generating and requires confirmation in adequately powered, multicenter studies before any clinical recommendation. Where available, Xpert MTB/RIF Ultra is the preferred first-line molecular assay and should be prioritized, in combination with T-SPOT.TB, in future prospective evaluation.

## 1 Introduction

Tuberculosis (TB) caused by *Mycobacterium tuberculosis* remains a global public-health emergency, with 10.8 million incident cases and 1.25 million deaths in 2023 (1). Pulmonary tuberculosis (PTB) is the predominant and most transmissible form. Paucibacillary PTB—PTB with a mycobacterial burden below the threshold for direct sputum-smear microscopy—disproportionately affects elderly patients, those with type 2 diabetes, and individuals with subtle immune dysregulation (2, 3), and is the form in which rapid diagnosis is hardest.

In paucibacillary PTB, sputum-smear microscopy achieves a sensitivity of only 30–40%, and liquid culture requires 2–8 weeks and biosafety infrastructure (4). The GeneXpert MTB/RIF assay gives same-day results, but its sensitivity falls from ∼66–70% in mixed bacteriological populations to below 43% in smear-negative specimens, because the rpoB-targeted assay requires an organism burden on the order of 10^2^–10^3^ CFU/mL (∼116 CFU/mL) for detection (5–7). IS6110 TB-DNA real-time PCR is limited by the same low-bacillary-load detection threshold as Xpert MTB/RIF, so its sensitivity in smear-negative disease is similarly constrained (8). The next-generation Xpert MTB/RIF Ultra assay has shown improved sensitivity (∼63–70%) in smear-negative specimens (6, 7, 9); however, Ultra was not available at our study site during the enrolment period, and its role in combination with IGRAs has not been prospectively evaluated.

T-SPOT.TB (Oxford Immunotec, United Kingdom) is a host immune-response assay—an interferon-γ release assay (IGRA) that enumerates ESAT-6– and CFP-10–specific effector-memory CD4^+^ T cells by ELISPOT. Because it measures the host immune response rather than the organism, its performance is independent of mycobacterial bacillary burden; pooled sensitivity for active PTB ranges 76–88% (10–12, 13).

These two test families occupy complementary diagnostic axes: molecular assays are highly specific rule-in/confirmatory tests that fail at low bacillary load, whereas T-SPOT.TB is a bacillary-load–independent test better suited to case-finding/triage when burden is low (14). This complementarity motivates the hypothesis that combining TB-DNA PCR with T-SPOT.TB could capture cases missed by either alone. Prospective data comparing such strategies specifically in the smear-negative paucibacillary context remain limited. We therefore characterized the individual and combined diagnostic accuracy of GeneXpert MTB/RIF, IS6110 TB-DNA PCR, and T-SPOT.TB, and explored which combination performed best, recognizing this as a single-center, hypothesis-generating evaluation.

## 2 Materials and methods

### 2.1 Study design and setting

**FIGURE 1:**
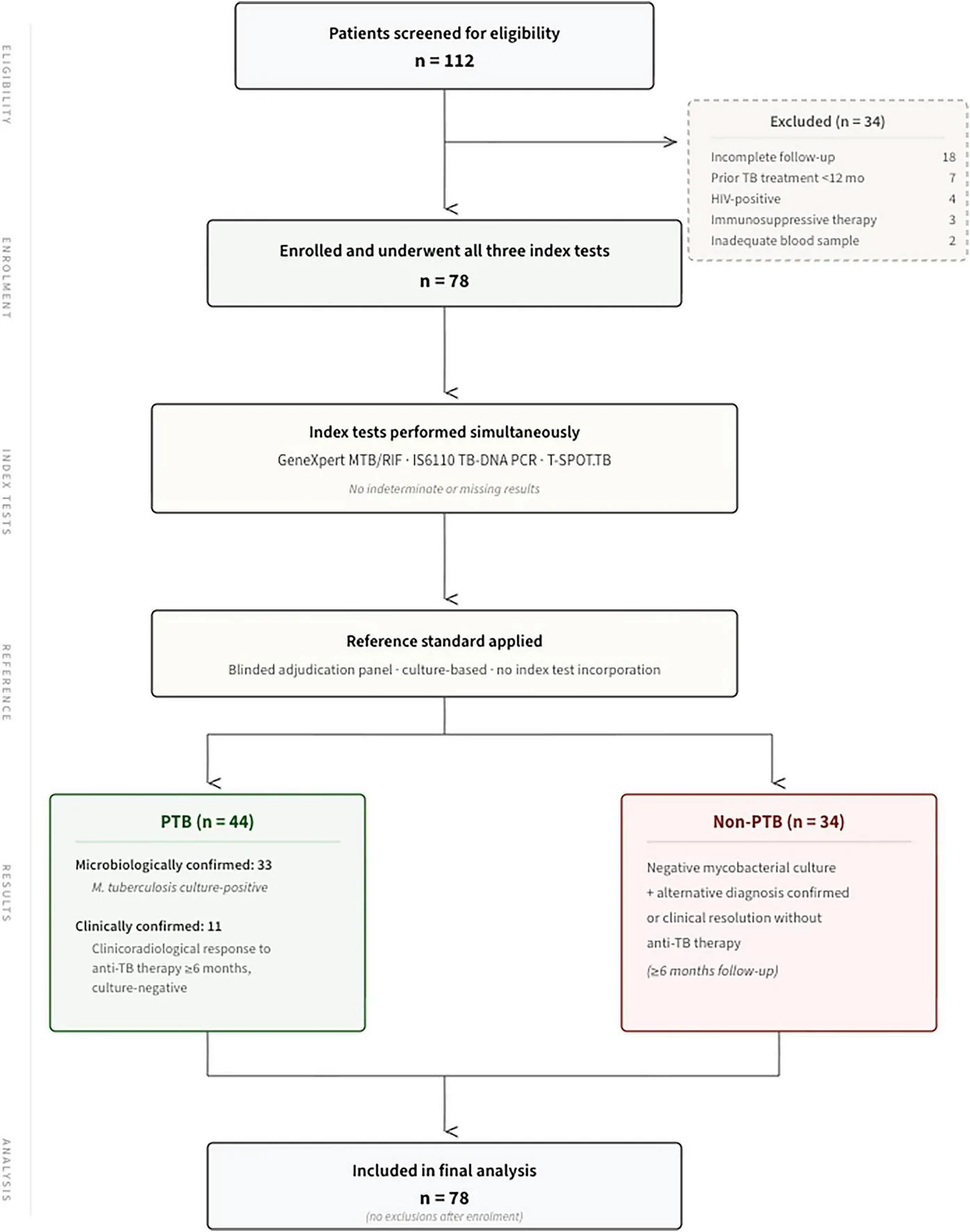
STARD 2015 participant flow diagram. 112 screened → 34 excluded (18 incomplete follow-up, 7 prior TB treatment, 4 HIV-positive, 3 immunosuppressive therapy, 2 inadequate blood sample) → 78 enrolled and tested by all three index tests. Final classification: PTB *n* = 44 (microbiological *n* = 33, clinical *n* = 11); non-PTB *n* = 34. No indeterminate/missing index-test results.

This single-center, prospective diagnostic-accuracy study was conducted in accordance with the STARD 2015 guidelines (15) at the Second Affiliated Hospital of Yichun University—a regional general hospital in Jiangxi Province, China—between January 2021 and December 2023. China is a WHO high-burden TB country [estimated national incidence ≈52 per 100,000 in 2023 (1)]; Jiangxi is an intermediate-burden province. The center provides routine TB diagnostics including smear microscopy, solid (Löwenstein–Jensen) and MGIT 960 liquid culture, GeneXpert MTB/RIF, an internally validated in-house IS6110 real-time PCR, and T-SPOT.TB. Xpert MTB/RIF Ultra was not in service during the enrolment window. Consecutive eligible patients presenting to outpatient or inpatient respiratory services were screened to minimize selection bias. The STARD flow diagram is shown in Figure 1.

### 2.2 Participants

Adults aged ≥ 18 years were eligible if they met all of: (a) ≥ 2 respiratory/constitutional symptoms suggestive of TB (cough, fever, weight loss, night sweats, or hemoptysis) persisting ≥ 2 weeks; (b) chest CT findings judged compatible with active PTB (e.g., centrilobular nodules, tree-in-bud, cavitation, or upper-lobe-predominant infiltrates); and (c) negative sputum-smear microscopy for acid-fast bacilli on ≥ 1 sample.

Patients were excluded if they had conditions known to substantially alter host immune response or microbiological yield: HIV seropositivity; current immunosuppressive therapy (systemic corticosteroids ≥ 10 mg/day prednisolone-equivalent for ≥ 14 days, or biological immunomodulators); prior anti-TB treatment within 12 months; inability to provide an adequate blood sample for T-SPOT.TB; or incomplete follow-up precluding reference-standard adjudication. Rationale for the immunosuppression exclusion: immunosuppression independently degrades both mycobacterial yield and IGRA responsiveness; immunocompetent patients were studied to establish baseline assay behavior without this confounder. The consequent limitation in generalizability is addressed in § 4.6.

The requirement for both compatible symptoms and CT findings creates a clinically enriched, high-pretest-probability cohort; consequently the PTB proportion observed here (56.4%) reflects enrolment enrichment, not background population prevalence, and predictive values should be interpreted accordingly (spectrum bias; §4.5). All participants were followed prospectively until a definitive diagnosis was established or anti-TB therapy completed.

#### 2.2.1 Definitions and case classification

Each of the 78 participants was classified as PTB or non-PTB by the blinded adjudication panel, applying the rule below identically to all participants:

- PTB—met either confirmation route:
- *Microbiological route:* positive *M. tuberculosis* culture from a respiratory specimen (see §2.3). Index-test results were not used in this route (incorporation-bias safeguard).
- *Clinical route:* culture-negative, but compatible clinicoradiological presentation plus documented clinical and radiological response to standard anti-TB therapy at 6 months, adjudicated by the panel.
- Non-PTB—met neither route: negative cultures, no response to empirical anti-TB therapy (where given), and an established alternative diagnosis (e.g., bacterial pneumonia, malignancy, sarcoidosis) or clinical resolution without anti-TB treatment over 6 months.

Precedence: any participant meeting the microbiological route was classified microbiologically, regardless of clinical course. Of 44 PTB cases, 33 met the microbiological route and 11 met the clinical route exclusively (all culture-negative). All 34 non-PTB participants met neither route.

### 2.3 Reference standard

The reference standard, applied independently of index-test results by a three-member panel (two respiratory physicians, one infectious-disease specialist) blinded to all index-test results, comprised the two PTB-confirmation routes defined in §2.2.1:

- Microbiological confirmation (*n* = 33): positive *M. tuberculosis* culture (Löwenstein–Jensen and/or MGIT 960) from sputum or BALF. Index tests (GeneXpert, TB-DNA PCR) were not used as confirmation criteria, avoiding incorporation bias.
- Clinical confirmation (*n* = 11): as defined in §2.2.1; culture-negative status was verified for all.

### 2.4 Index tests

All index tests were performed during the same clinical visit to avoid temporal bias. Laboratory personnel performing each assay were blinded to the other index tests and to the final diagnosis.

GeneXpert MTB/RIF. Respiratory specimens (spontaneous sputum or BALF) were processed according to the manufacturer’s instructions (Cepheid, Sunnyvale, CA, United States). Xpert MTB/RIF results were interpreted using the four semiquantitative categories generated by the classic cartridge: high, medium, low, and very low. A result was classified as positive when Mycobacterium tuberculosis complex was detected in any of these categories; otherwise, it was classified as negative. Xpert MTB/RIF Ultra was not used in this study, and Ultra-specific “trace” calls were therefore not generated, recorded, or included in the analysis.

TB-DNA PCR (IS6110 real-time PCR). The same respiratory specimen used for GeneXpert was analysed using an internally validated real-time quantitative PCR targeting the IS6110 insertion sequence, performed on DNA extracted from the respiratory specimen (not artificially purified template). The detection threshold was ∼116 CFU/mL; cycle-threshold (Ct) values ≤ 38 were predefined as positive based on internal validation.

T-SPOT.TB (ELISPOT). PBMCs were isolated and tested per the manufacturer’s protocol (Oxford Immunotec, United Kingdom); 2.5 × 105 PBMCs were plated per well and stimulated with ESAT-6 and CFP-10 antigens. A result was positive if either antigen well showed ≥ 6 spot-forming cells above the negative control.

### 2.5 Clinical risk score (exploratory)

A predefined Clinical Risk Score (CRS; five equally weighted variables—cough ≥ 2 weeks, fever ≥ 38 °C, unintentional weight loss > 3 kg, haemoptysis, and CT features of active TB; range 0–5; CRS ≥ 2 = high-risk) was assigned at enrolment. Because the CRS did not materially alter the primary findings, it is reported only as a baseline characteristic (Table 1) and not analyzed further.

**TABLE 1:** Baseline demographic and clinical characteristics (*n* = 78).

Characteristic	All (*n* = 78)	PTB (*n* = 44)	Non-PTB (*n* = 34)	*P*
Age, years (mean ± SD)	48.4 ± 11.4	49.9 ± 11.5	46.3 ± 11.2	0.169
Male sex, n (%)	54 (69.2)	31 (70.5)	23 (67.6)	0.776
Cough ≥ 2 weeks, n (%)	72 (92.3)	42 (95.5)	30 (88.2)	0.258
Fever ≥ 38 °C, n (%)	52 (66.7)	32 (72.7)	20 (58.8)	0.179
Weight loss > 3 kg, n (%)	30 (38.5)	25 (56.8)	5 (14.7)	< 0.001
Haemoptysis, n (%)	9 (11.5)	9 (20.5)	0 (0.0)	0.014
Diabetes mellitus, n (%)	14 (17.9)	9 (20.5)	5 (14.7)	0.499
CRP, mg/L (median, IQR)	8.6 (5.4–13.0)	10.6 (6.0–15.0)	6.2 (4.8–8.0)	0.001
CRS ≥ 2, n (%)	31 (39.7)	27 (61.4)	4 (11.8)	< 0.001
CT tree-in-bud, n (%)	24 (30.8)	18 (40.9)	6 (17.6)	0.050
CT cavitation, n (%)	14 (17.9)	9 (20.5)	5 (14.7)	0.499

CRS, Clinical Risk Score; CRP, C-reactive protein; IQR, interquartile range; SD, standard deviation.

### 2.6 Sample-size considerations

No a priori sample-size calculation was performed. Enrolment was determined by consecutive patient availability over a fixed 3-year window. A *post-hoc* estimate indicates that with 44 PTB cases the study has ∼80% power to detect a ∼25-percentage-point sensitivity difference between paired assays (two-sided McNemar, α = 0.05); this estimate is descriptive only and does not validate the design. Subgroup analyses (specimen type and confirmation route) are exploratory, with correspondingly wide confidence intervals. All analyses in this study are therefore exploratory and hypothesis-generating (see § 4.6).

### 2.7 Statistical analysis

Categorical variables are presented as counts and percentages with 95% confidence intervals (Wilson method). Continuous variables are mean ± SD (normal) or median (IQR). Group comparisons used the χ^2^ or Fisher exact test (categorical) and Student t or Mann–Whitney U test (continuous). Diagnostic performance was summarized by sensitivity, specificity, PPV, NPV, LR+, LR-, and balanced accuracy = (sensitivity + specificity)/2, with 95% CIs by bootstrap percentile (5,000 resamples). Balanced accuracy equals the area under the three-point ROC curve through (0,0), (FPR, TPR), and (1,1) for a binary classifier. Paired comparisons used the McNemar exact test; to account for the seven strategies (three individual, four combination), Bonferroni correction was applied (adjusted *p* < 0.05 significant).

## 3 Results

### 3.1 Participant flow and baseline characteristics

Of 112 patients screened, 34 were excluded (18 incomplete follow-up, 7 prior TB treatment, 4 HIV-positive, 3 immunosuppressive therapy, 2 inadequate blood sample), leaving 78 participants (Figure 1). Of these, 44/78 (56.4%) were classified PTB (33 microbiological, 11 clinical) and 34/78 (43.6%) non-PTB.

Mean age was 48.4 ± 11.4 years (PTB 49.9 ± 11.5 vs. non-PTB 46.3 ± 11.2; *p* = 0.169). The PTB group had higher rates of weight loss (56.8% vs. 14.7%; *p* < 0.001), haemoptysis (20.5% vs. 0.0%; *p* = 0.014), elevated CRP (median 10.6 vs. 6.2 mg/L; *p* = 0.001), CRS ≥ 2 (61.4% vs. 11.8%; *p* < 0.001), and CT tree-in-bud (40.9% vs. 17.6%; *p* = 0.050). Baseline characteristics are in Table 1.

### 3.2 Individual assay performance

**FIGURE 2:**
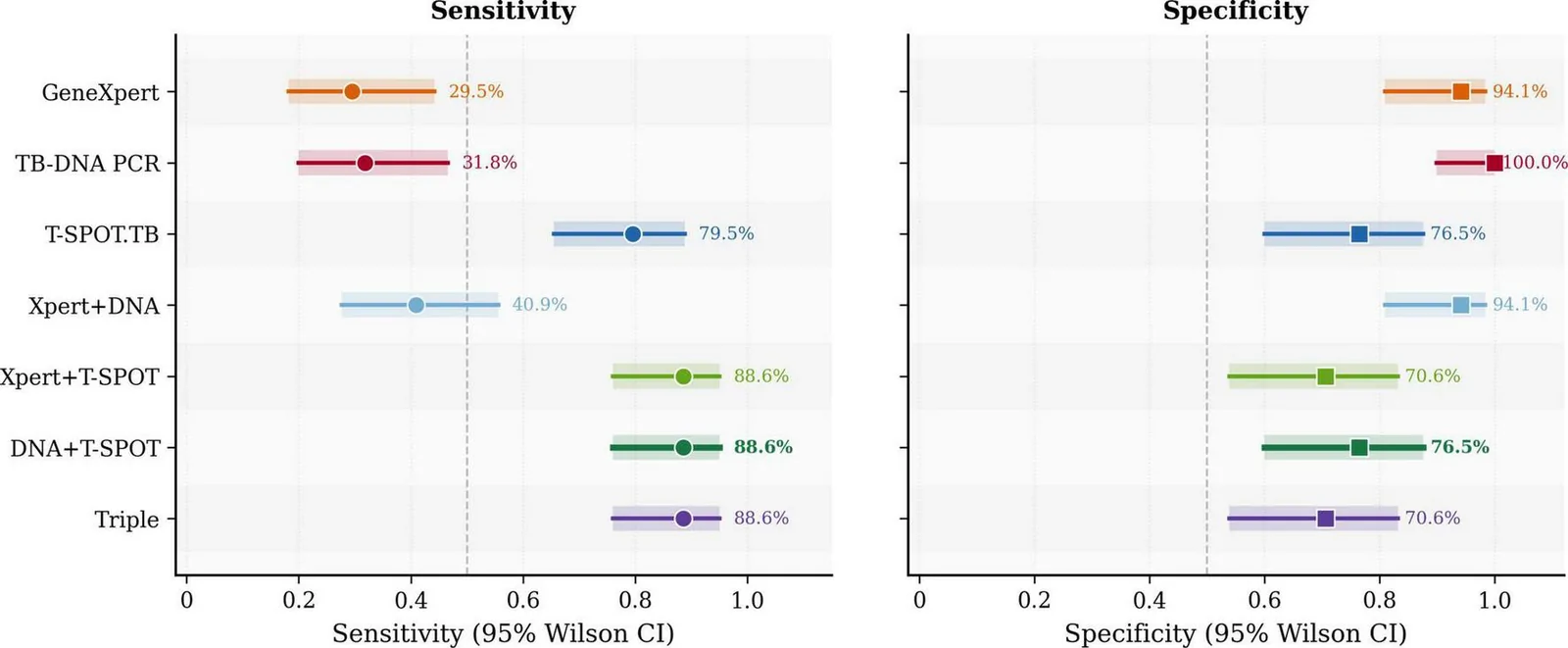
Sensitivity (left) and specificity (right) with 95% Wilson CIs for individual and combined strategies.

Individual performance is in Figure 2 and Table 2. GeneXpert detected 13/44 PTB cases (sensitivity 29.5%, 95% CI 18.2–44.2%; specificity 94.1%, 80.9–98.4%; PPV 86.7%; NPV 50.8%; balanced accuracy 0.618 [0.541–0.698]; LR+ 5.02). TB-DNA PCR detected 14/44 (sensitivity 31.8%, 20.0–46.6%; specificity 100.0%, 89.8–100.0%; PPV 100.0%; NPV 53.1%; balanced accuracy 0.659 [0.591–0.729]; LR+ ∞, zero false positives). T-SPOT.TB detected 35/44 (sensitivity 79.5%, 65.5–88.8%; specificity 76.5%, 60.0–87.6%; PPV 81.4%; NPV 74.3%; balanced accuracy 0.780 [0.681–0.872]; LR+ 3.38, LR- 0.27).

**TABLE 2:** Individual assay diagnostic performance (*n* = 78; PTB *n* = 44, non-PTB *n* = 34).

Assay	TP/FP/FN/TN	Sens % (95% CI)	Spec % (95% CI)	Bal. Acc. (95% CI)	PPV %	NPV %	LR+
GeneXpert	13/2/31/32	29.5 (18.2–44.2)	94.1 (80.9–98.4)	0.618 (0.541–0.698)	86.7	50.8	5.02
TB-DNA PCR	14/0/30/34	31.8 (20.0–46.6)	100.0 (89.8–100.0)	0.659 (0.591–0.729)	100.0	53.1	∞
T-SPOT.TB	35/8/9/26	79.5 (65.5–88.8)	76.5 (60.0–87.6)	0.780 (0.681–0.872)	81.4	74.3	3.38

T-SPOT.TB sensitivity was significantly superior to GeneXpert (exact McNemar *p* < 0.001) and TB-DNA PCR (*p* < 0.001); both remained significant after Bonferroni correction.

#### 3.2.1 Confirmation-route distribution and interpretive context

Among the 44 confirmed PTB cases, 33 (75.0%) were microbiologically confirmed and 11 (25.0%) were clinically confirmed exclusively. Because the clinically confirmed subgroup was small and culture-negative by definition, route-specific diagnostic-performance estimates would be unstable and were not used to support additional comparative claims. The manuscript therefore presents the confirmation-route distribution explicitly and interprets the low molecular sensitivity in the context of paucibacillary, smear-negative disease rather than as a route-specific diagnostic claim.

### 3.3 Combination strategies

**FIGURE 3:**
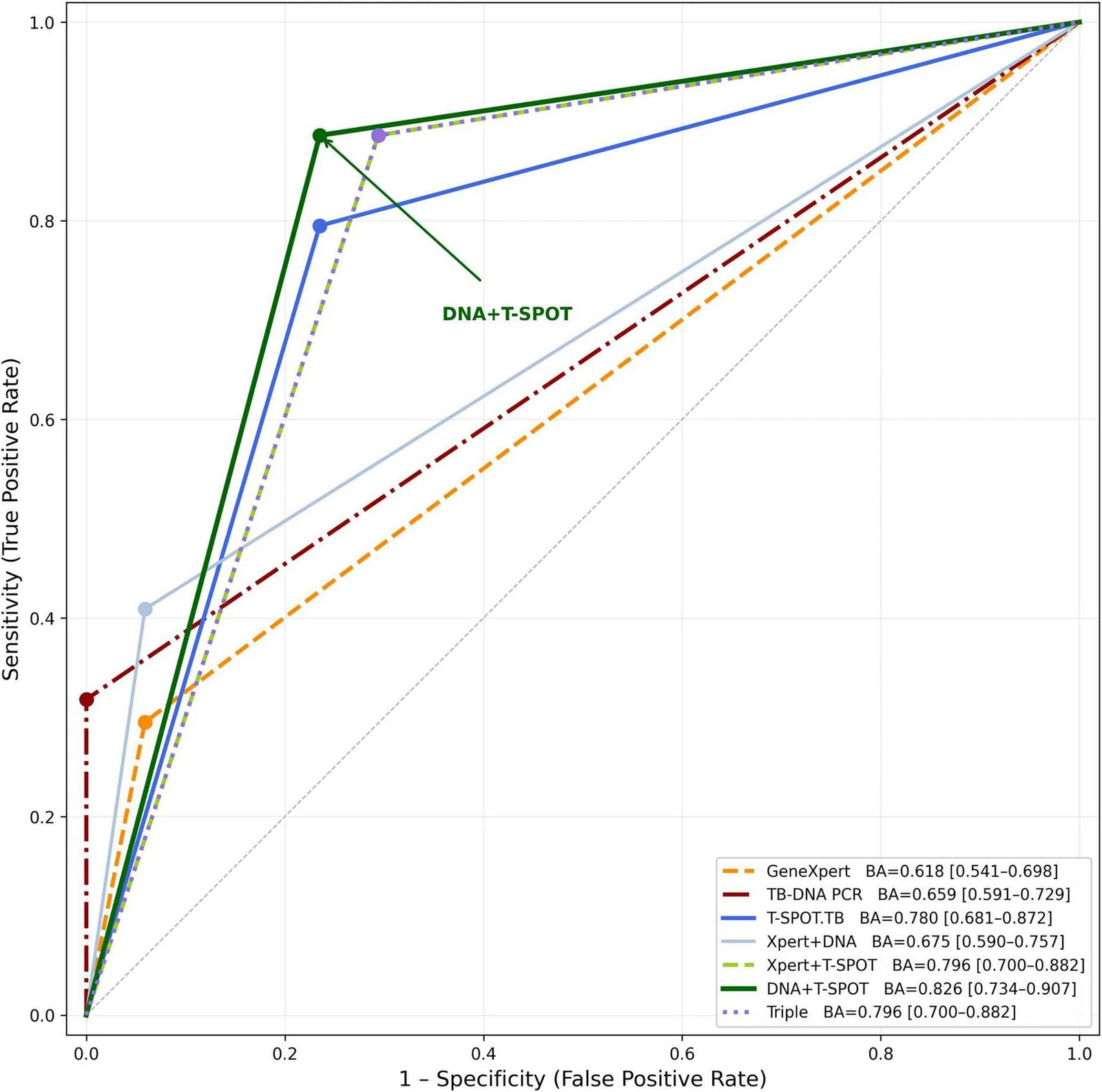
Diagnostic operating points for individual and combined strategies in ROC space; each strategy is a single binary operating point connected to (0,0) and (1,1). Balanced accuracy = area under the three-point ROC curve. TB-DNA + T-SPOT.TB highest (0.826; 0.734–0.907).

Figure 3 and Table 3 present combinations under a parallel (any-positive) rule. GeneXpert + TB-DNA improved sensitivity to 40.9% (18/44) but its sensitivity remained significantly lower than that of T-SPOT.TB alone (79.5%; exact McNemar p < 0.001), and its balanced accuracy was 0.675.

**TABLE 3:** Combined assay performance under parallel (any-positive) decision rule.

Combination	TP/FP/FN/TN	Sens % (95% CI)	Spec % (95% CI)	Bal. Acc. (95% CI)	PPV %	NPV %	LR+	p vs. T-SPOT
GeneXpert + TB-DNA	18/2/26/32	40.9 (27.7–55.6)	94.1 (80.9–98.4)	0.675 (0.590–0.757)	90.0	55.2	6.95	< 0.001
GeneXpert + T-SPOT.TB	39/10/5/24	88.6 (76.0–95.0)	70.6 (53.8–83.2)	0.796 (0.700–0.882)	79.6	82.8	3.01	0.125
TB-DNA + T-SPOT.TB	**39/8/5/26**	**88.6 (76.0–95.0)**	**76.5 (60.0–87.6)**	**0.826 (0.734–0.907)**	**83.0**	**83.9**	**3.77**	**0.125**
Triple	39/10/5/24	88.6 (76.0–95.0)	70.6 (53.8–83.2)	0.796 (0.700–0.882)	79.6	82.8	3.01	0.125

Bold indicates the highest observed balanced accuracy in this cohort. p: two-sided exact McNemar test of sensitivity versus T-SPOT.TB alone (uncorrected). For each T-SPOT.TB–containing combination the discordant pairs are one-directional (the combination only adds positives to T-SPOT.TB, so c = 0); all three therefore yield the same exact *p* = 0.125 and none is statistically significant, a conclusion unchanged by Bonferroni correction.

TB-DNA PCR + T-SPOT.TB showed the highest balanced accuracy in this cohort: sensitivity 88.6% (39/44; 76.0–95.0%), specificity 76.5% (26/34; 60.0–87.6%), PPV 83.0%, NPV 83.9%, balanced accuracy 0.826 (0.734–0.907), LR+ 3.77, LR- 0.15. Versus T-SPOT.TB alone, sensitivity rose from 79.5 to 88.6%; because the four discordant pairs all favored the combination (*b* = 4, *c* = 0), the exact two-sided McNemar *p* = 0.125 and this increase did not reach statistical significance. Specificity was unchanged (76.5%) because TB-DNA’s 100% specificity adds no false positives.

GeneXpert + T-SPOT.TB gave balanced accuracy 0.796 (0.700–0.882; specificity reduced to 70.6% by GeneXpert’s 2 additional false positives). The balanced-accuracy difference versus TB-DNA + T-SPOT.TB (0.826 vs. 0.796) corresponds to only 2/34 non-PTB patients and warrants cautious interpretation. The triple combination gave balanced accuracy 0.796—identical to GeneXpert + T-SPOT.TB—indicating no incremental discriminatory benefit from adding GeneXpert to the TB-DNA PCR + T-SPOT.TB dual strategy.

### 3.4 Venn analysis

**FIGURE 4:**
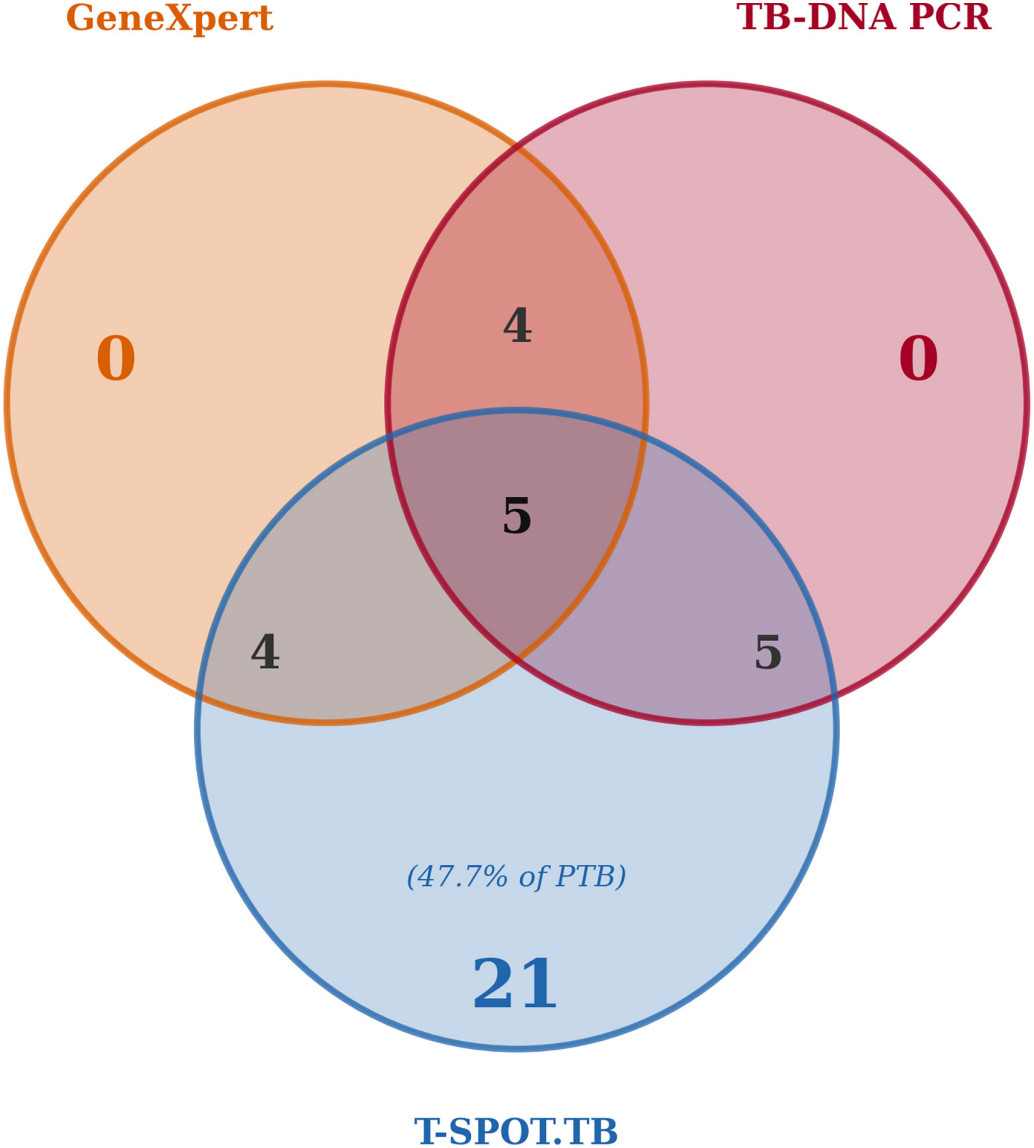
Venn diagram of assay positivity among 44 confirmed PTB cases; T-SPOT.TB sole positive in 21/44 (47.7%); neither molecular assay exclusively detected any case; 5/44 (11.4%) undetected by all three (not shown).

Among 44 PTB cases (Figure 4), T-SPOT.TB was the sole positive assay in 21/44 (47.7%)—the largest exclusive contribution of any assay. GeneXpert and TB-DNA PCR had 0 exclusive detections, confirming complete overlap between the molecular assays. Overlaps: GeneXpert ∩ TB-DNA only 4/44 (9.1%); GeneXpert ∩ T-SPOT only 4/44 (9.1%); TB-DNA ∩ T-SPOT only 5/44 (11.4%); all three positive 5/44 (11.4%). Five cases (11.4%) were undetected by all three assays.

### 3.5 Subgroup analyses (exploratory)

**FIGURE 5:**
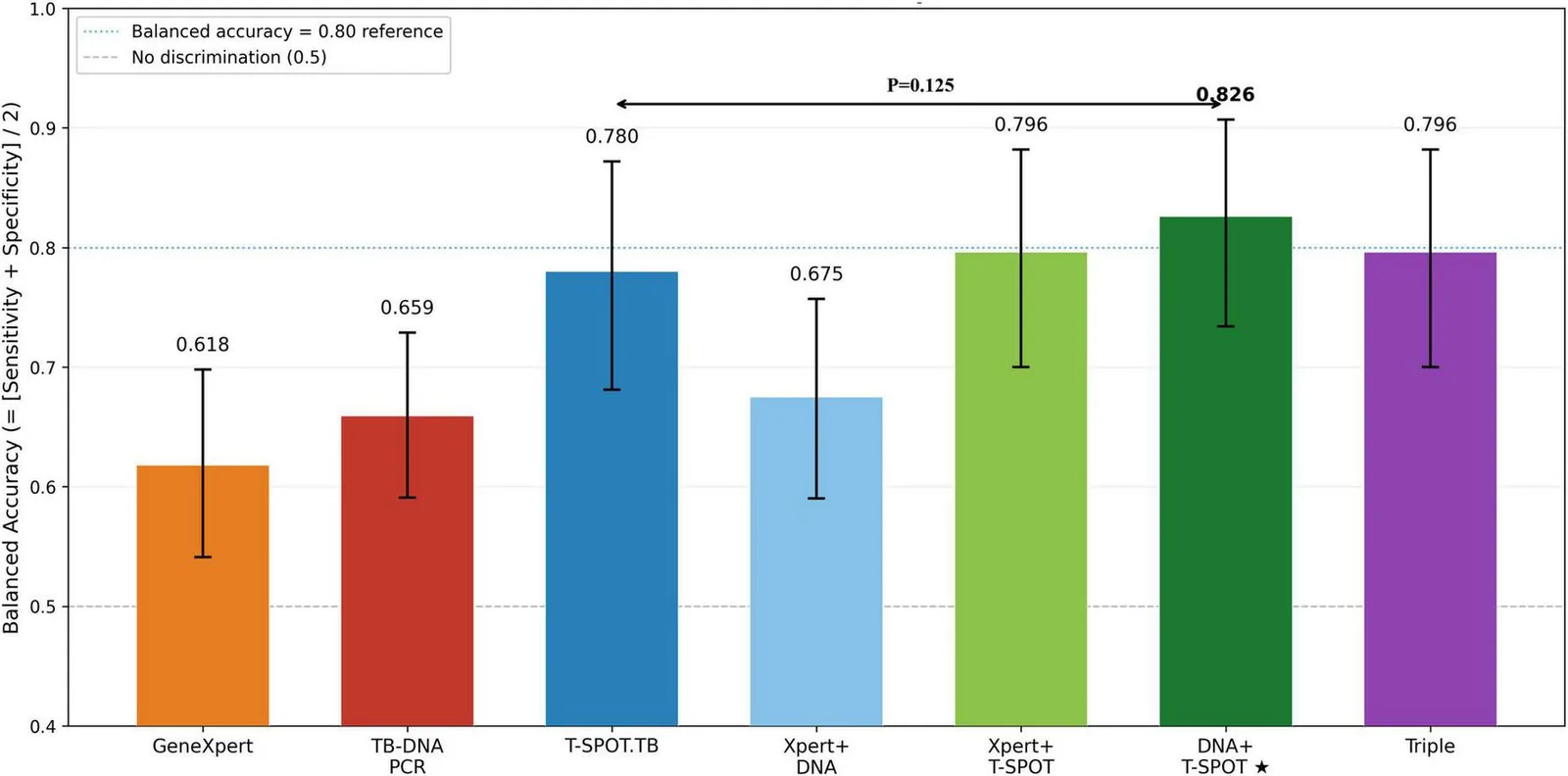
Balanced-accuracy values with 95% bootstrap CIs for all strategies; reference line at 0.80.

Subgroup data are in Table 4. In BALF (PTB *n* = 11), molecular sensitivities were modestly higher than in sputum (GeneXpert 36.4% vs. 28.1%; TB-DNA 36.4% vs. 31.2%), consistent with higher bronchoscopic yield; T-SPOT.TB was 72.7% (BALF) vs. 81.2% (sputum). One further PTB case had a specimen recorded as “other” and is listed separately in Table 4 (11 + 32 + 1 = 44). All subgroup estimates carry wide CIs and are hypothesis-generating only. Figure 5 shows balanced-accuracy comparisons across strategies.

**TABLE 4:** Sensitivity by specimen type (exploratory subgroups).

Subgroup	GeneXpert (95% CI)	TB-DNA (95% CI)	T-SPOT.TB (95% CI)	TB-DNA + T-SPOT.TB (95% CI)
Overall (PTB *n* = 44)	29.5 (18.2–44.2)	31.8 (20.0–46.6)	79.5 (65.5–88.8)	88.6 (76.0–95.0)
BALF (PTB *n* = 11)	36.4 (15.2–64.6)	36.4 (15.2–64.6)	72.7 (43.4–90.3)	90.9 (62.3–98.4)
Sputum (PTB *n* = 32)	28.1 (15.6–45.0)	31.2 (17.6–49.2)	81.2 (64.7–91.4)	87.5 (71.9–95.2)
Other (PTB *n* = 1)†	0.0 (–)	0.0 (–)	100.0 (–)	100.0 (–)

All subgroup estimates carry wide CIs and are hypothesis-generating. †One PTB case had a specimen type recorded as “other”; confidence intervals are not estimated for a single case.

## 4 Discussion

This single-center, exploratory prospective study of smear-negative paucibacillary PTB (*n* = 78) yields four principal observations, which we frame as hypotheses for confirmation: (i) T-SPOT.TB was the most informative individual assay (sensitivity 79.5%; balanced accuracy 0.780; *p* < 0.001 vs. molecular assays); (ii) TB-DNA PCR + T-SPOT.TB showed the highest observed dual-combination balanced accuracy (0.826; sensitivity gain over T-SPOT.TB alone not statistically significant, exact McNemar *p* = 0.125); (iii) the triple combination did not improve discrimination (0.796 vs. 0.826); and (iv) T-SPOT.TB was the sole positive assay in 47.7% of confirmed PTB cases.

### 4.1 Molecular sensitivity is constrained by bacillary load

The low molecular sensitivity (GeneXpert 29.5%; TB-DNA 31.8%) is mechanistically expected: IS6110 PCR requires ∼116 CFU/mL, exceeding the typical burden of smear-negative sputum. Cochrane analyses (7, 16) report GeneXpert sensitivity ∼43% in smear-negative specimens, consistent with our results.

### 4.2 Xpert versus in-house IS6110 PCR

The near-identical sensitivity of GeneXpert (29.5%) and in-house IS6110 qPCR (31.8%) is best explained by a shared analytical floor: both are nucleic-acid amplification tests limited by the same order-of-magnitude limit of detection (∼10^2^–10^3^ CFU/mL), which exceeds the burden of most smear-negative specimens. The convergence therefore reflects the biological ceiling imposed by low bacillary load, not suboptimal in-house performance—supported by the in-house assay’s 100% specificity (0/34 false positives) and prior head-to-head IS6110-PCR-versus-Xpert data (8). The in-house assay operated on DNA extracted from respiratory specimens, not idealized purified template; its perfect specificity makes each positive result a near-definitive rule-in.

### 4.3 Why combine modalities, and is the gain meaningful?

The two modalities serve complementary clinical roles: molecular assays are highly specific rule-in/confirmatory tests, whereas T-SPOT.TB is a bacillary-load–independent case-finding/triage test. Combining them under a parallel rule is a deliberate sensitivity-maximizing strategy for the specific decision point of smear-negative patients in whom anti-TB therapy is being considered but confirmation is lacking — not a general screening recommendation. The dual TB-DNA + T-SPOT.TB strategy raised sensitivity from 79.5 to 88.6% (exact McNemar *p* = 0.125; not statistically significant) without lowering specificity (76.5%), a relatively favorable trade-off, because TB-DNA’s 100% specificity introduces no false positives; by contrast, Xpert-containing parallel combinations lowered specificity (to 70.6%) for no sensitivity gain. Given the wide CIs and the absence of a priori powering (§4.6), the 9-point sensitivity gain should be regarded as a hypothesis to be tested, and in practice parallel testing must be weighed against the over-treatment cost of additional false positives in the local prevalence context.

### 4.4 T-SPOT.TB in paucibacillary disease

The diagnostic primacy of T-SPOT.TB reflects immunological independence from bacillary burden (17, 18). Our sensitivity (79.5%) and 47.7% exclusive-detection rate are consistent with pooled IGRA estimates of 76–88% (13, 19–21). An important limitation in our BCG-vaccinated Chinese population is the specificity of only 76.5%: the 8 false positives among 34 non-PTB controls likely represent latent TB infection (LTBI) (22, 23). In lower-LTBI settings, specificity may be higher; in very-high-burden settings, the false-positive rate may rise, reducing utility. Critically, because an IGRA detects immunological memory of *M. tuberculosis* sensitization and cannot by itself distinguish active disease from latent infection, a positive T-SPOT.TB in a paucibacillary patient must not be interpreted in isolation: it cannot serve as a stand-alone confirmatory test or an independent diagnostic pillar, and must be integrated with clinical, radiological, and epidemiological context—as enforced in this study by the composite reference standard and blinded panel adjudication.

### 4.5 Generalizability, setting, and the role of Xpert Ultra

This was a single-center study in an intermediate-burden Chinese province, using a clinically enriched high-pretest-probability cohort; predictive values are conditioned on this enrichment and the single-center case mix, and may not transfer to other settings or to unselected screening populations.

We emphasize that where Xpert MTB/RIF Ultra is accessible, its higher reported sensitivity in smear-negative disease (∼63–70%) and its lower per-patient cost and technical demand relative to ELISPOT-based IGRA make it the rational first-line molecular test; a triple-test approach cannot be recommended as a routine default. Our findings should be read as evidence on the *complementary immunological contribution of T-SPOT.TB* where the available molecular assay remains insensitive, rather than as an endorsement of universal triple testing. Ultra was not available at our site during enrolment; had it been used, the sensitivity of Xpert-containing strategies would likely have been higher, potentially altering the performance hierarchy. Direct prospective evaluation of Xpert Ultra + T-SPOT.TB is the priority next step.

### 4.6 Limitations

First, no a priori sample-size calculation was performed; the study can exclude only large differences and is not powered for the smaller, clinically nuanced differences (e.g., the specificity contrasts between combinations) discussed here. Accordingly, all between-strategy differences are hypothesis-generating, and the conclusions require confirmation in adequately powered multicenter studies.

Second, the composite reference standard, while necessary given culture-negative TB, risks misclassification: the 11 clinically confirmed cases could include non-TB patients who responded coincidentally; this was mitigated by requiring 6-month documented response and blinded adjudication, but residual misclassification cannot be excluded. Because culture (not molecular tests) served as the microbiological route, incorporation bias does not affect molecular sensitivity estimates; however, partial verification dependency may modestly inflate molecular specificity.

Third, exclusion of HIV-positive and immunosuppressed patients limits generalizability to precisely the populations in whom paucibacillary smear-negative disease is most common and consequential. T-SPOT.TB sensitivity in particular falls under CD4 depletion; our 79.5% individual and 88.6% combined sensitivities therefore likely overestimate performance in immunocompromised cohorts. Evaluation in these populations is a priority.

Fourth, the single-center design in a BCG-vaccinated, intermediate-burden Chinese setting limits applicability to other epidemiological contexts. Fifth, balanced accuracy weights sensitivity and specificity equally; clinicians may prioritize one depending on context.

## 5 Conclusion

In this single-center, exploratory study of smear-negative paucibacillary PTB, T-SPOT.TB contributed unique diagnostic information (47.7% exclusive detection) not captured by molecular assays. TB-DNA PCR + T-SPOT.TB showed the highest observed balanced accuracy (0.826; sensitivity 88.6%, specificity 76.5%); however, the sensitivity advantage over T-SPOT.TB alone did not reach statistical significance (exact McNemar *p* = 0.125), and—given the single-center design and the absence of a priori powering—this finding is hypothesis-generating and must be confirmed in adequately powered, multicenter cohorts before any clinical recommendation. Where available, Xpert MTB/RIF Ultra is the preferred first-line molecular assay; prospective evaluation of Xpert Ultra + T-SPOT.TB, including in HIV-positive and immunosuppressed populations, is the priority for future work.

## Data Availability

The raw data supporting the conclusions of this article will be made available by the authors, without undue reservation.
